# Hybrid surgery recanalization for high-level chronic internal carotid artery occlusion

**DOI:** 10.3389/fneur.2023.1161277

**Published:** 2023-06-21

**Authors:** Yuankun Cai, Tingbao Zhang, Lesheng Wang, Xiang Li, Wei Wei, Yu Feng, Guo Li, Yihui Ma, Xinjun Chen, Wenyuan Zhao, Jincao Chen

**Affiliations:** Department of Neurosurgery, Zhongnan Hospital of Wuhan University, Wuhan, Hubei, China

**Keywords:** chronic internal carotid artery occlusion, hybrid surgery, carotid endarterectomy (CEA), carotid artery stent (CAS), recanalization

## Abstract

**Objective:**

Although endovascular recanalization is considered a more effective treatment for chronic internal carotid artery occlusion (CICAO), the success rate of complex CICAO remains inadequate. We present hybrid surgery (carotid endarterectomy combined with carotid stenting) for complex CICAO and explore the influential factors and effects of hybrid surgery recanalization.

**Methods:**

We retrospectively analyzed the clinical, imaging, and follow-up data of 22 patients with complex CICAO treated by hybrid surgery at the Zhongnan Hospital of Wuhan University from December 2016 to December 2020. We also summarize the technical points related to hybrid surgery recanalization.

**Results:**

A total of 22 patients with complex CICAO underwent hybrid surgery recanalization. There were no postoperative deaths in all patients after hybrid surgery recanalization. Nineteen patients successfully underwent recanalization with a success rate of 86.4% and three cases with a failure rate of 13.6%. Patients were divided into success and failure groups. Significantly different radiographic classification of lesions was observed between the success group and the failure group (*P* = 0.019). The rates of CICAO with reverse ophthalmic artery blood flow in the internal carotid artery (ICA) preoperatively were 94.7% in the success group and 33.3% in the failure group (*P* = 0.038). Three cases of hybrid surgery recanalization failure were transferred for EC-IC bypass and had good neurological recovery. Postoperative average KPS scores of the 19 patients were improved compared to the preoperative ones (*P* < 0.001).

**Conclusion:**

Hybrid surgery for complex CICAO is safe and effective with a high recanalization rate. The recanalization rate is related to whether the occluded segment surpasses the ophthalmic artery.

## Introduction

Chronic internal carotid artery occlusion (CICAO) is characterized by progressive bilateral or unilateral occlusion of the internal carotid artery for more than 4 weeks, which carries higher recurrence rates of stroke, disability, and mortality (1). The main treatment modalities include medical therapy (antiplatelet and anticoagulant), extracranial–intracranial (EC-IC) bypass, carotid endarterectomy (CEA), and endovascular revascularization. The overall risk of stroke from CICAO is ~5–7% per year despite receiving the best available medical therapy (2–4). A carotid occlusion surgery study (COSS) in 2011 (5) indicated that EC-IC bypass surgery could improve the hemodynamic indicators of patients to some extent but could not effectively reduce the stroke recurrence rate. Furthermore, studies have shown that CEA is only suitable for short-segment occlusion limited to the neck (the success rate of opening occlusions was only 18.5–40.2%, and the postoperative restenosis rate was as high as 30.7–76.6%) (6, 7). With the development of endovascular intervention techniques and changes in the materials used, endovascular revascularization for CICAO is gradually being promoted. In 2018, Hasan et al. proposed a high success rate of open intravascular intervention of type A and B CICAO but a low success rate for cumulative carotid bifurcation of type C and D CICAO of the ophthalmic artery (8, 9).

The application of hybrid surgery (CEA combined with internal carotid artery stent implantation (CAS) in the same period) for the recanalization of CICAO has been reported in recent years and has achieved good outcomes (10–12). However, factors that affect the rate of revascularization and improvement of the daily living ability of patients have yet to be fully reported. This study aimed to explore the effects of hybrid surgery for complex CICAO (type C and D CICAO), describe the relevant technical points, and analyze factors that affect the recanalization rate.

## Materials and methods

### Patient and data collection

**Figure 1:**
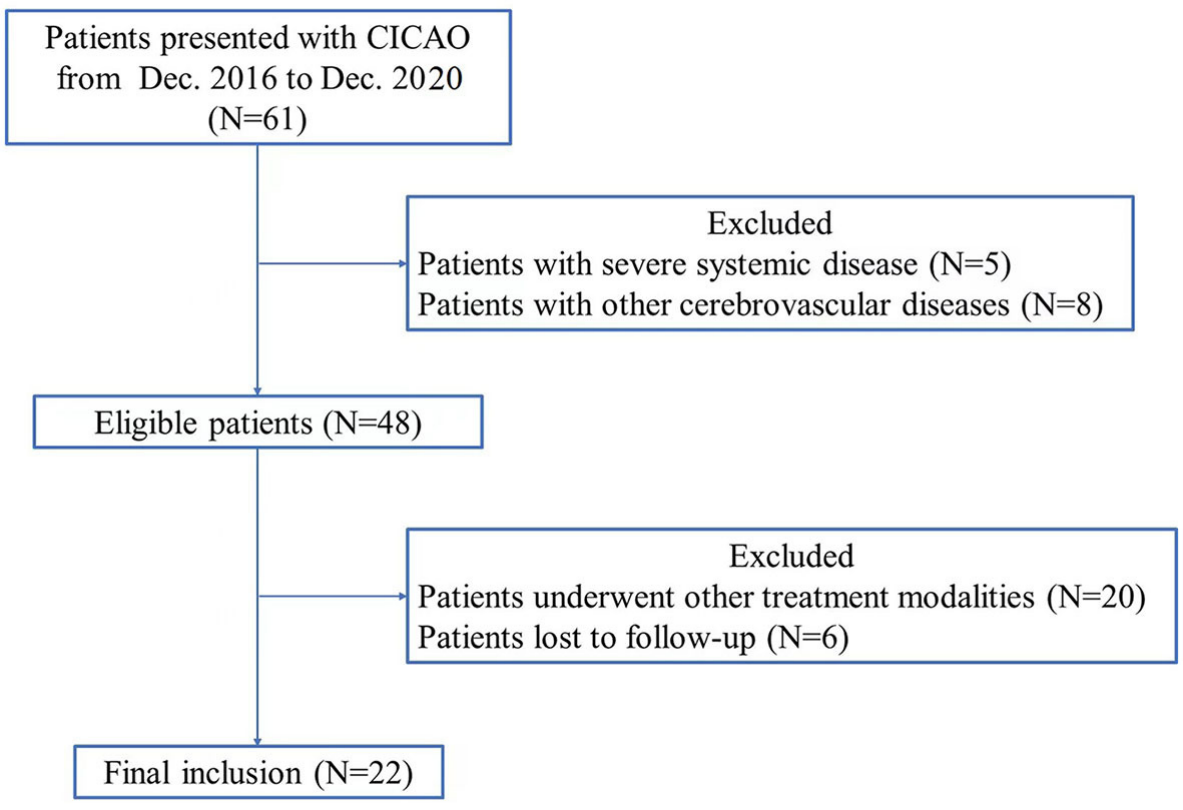
Flowchart of patient inclusion and exclusion.

Ethics approval was waived by the local Ethics Committee of Wuhan University in view of the retrospective nature of the study, and all the procedures that were performed were part of routine care. We retrospectively analyzed the clinical, imaging, and follow-up data of CICAO patients treated by hybrid surgery from December 2016 to December 2020 (Figure 1). CICAO was defined as a duration of ≥4 weeks between diagnosis and treatment. The inclusion criteria were as follows: (i) those with complete occlusion of one side of the internal carotid artery (i.e., the occluded segment is larger than the cervical segment and the duration of ICA occlusion is more than 4 weeks); (ii) those with related clinical neurological symptoms; (iii) those with internal carotid artery occlusion who underwent hybrid surgery for recanalization, excluding those of simple CEA and CAS recanalization; and (iv) based on the newly suggested radiographic classification proposed by Hasan et al. (8), Type C CICAO with no ICA stump and patent lumen distally with collateral filling, from branches of the ECA, Pcom, and/or ACA, and Type D CICAO with no ICA stump and occluded lumen distally until the ICA bifurcation.

Exclusion criteria were listed as follows: (i) acute or subacute CICAO; (ii) other cerebrovascular diseases (Moyamoya disease, intracranial aneurysms, and cerebrovascular malformations) were excluded; (iii) the presence of severe systemic diseases in the heart, liver, kidney, lung, or other vital organs; (iv) inability to tolerate general anesthesia for surgery; and (v) patients who underwent other treatment modalities including pharmacological treatment, simple CEA or CAS recanalization, and intracranial and extracranial revascularization.

Demographic and baseline data included age, sex, smoking, past medical history (diabetes, hypertension, and heart disease), and duration of occlusion. The Karnofsky Performance Scale (KPS) scores on admission and duration from the last neurological event to surgery were also collected. Two neurosurgeons assessed all the images. The occlusion length, reverse ocular artery blood flow in ICA preoperatively, and collateral circulation measured by digital subtraction angiography (DSA) and cerebral blood perfusion (CTP) were collected and analyzed.

### Preoperative preparations

Preoperative imaging and laboratory examinations were performed to eliminate surgical contraindications and evaluate the success rate and safety of the operation. All patients underwent neck vascular ultrasonography (US), a transcranial Doppler (TCD) study, magnetic resonance imaging (MRI), cerebrovascular DSA, computed tomography for the assessment of CTP or MRI for the evaluation of cerebral blood perfusion (PWI), cardiac US, and other related imaging examinations preoperatively and at 6 months postoperatively. All patients completed high-resolution MRI (HRMRI) for plaque examination and analysis.

Patients were prescribed oral aspirin (100 mg/day), clopidogrel sulfate (75 mg/day), and an atorvastatin calcium tablet (20 mg/night) for at least 1 week. If the patient showed resistance to clopidogrel hydrogen sulfate based on the chromogram and platelet aggregation rate, it was replaced with an oral ticagrelor tablet (90 mg/day for 2–3 days), and the platelet aggregation rate was rechecked to ensure that the inhibition rate was <50%.

### Operative process

After successful general anesthesia and electrophysiological monitoring, the patient was placed in the supine position. After a successful femoral artery puncture using the right inguinal Seldinger technique, an 8-Fr artery sheath was placed, and the lesion location, occlusion length, occlusion type, and occlusion compensation were re-examined using angiography. After systemic heparinization, the guiding catheter was placed below the carotid artery bifurcation and adequately fixed.

The anterior approach to the sternocleidomastoid muscle on the side of the lesion was used after fixation was confirmed. The incision length was adjusted according to the bifurcation of the carotid artery. An incision of the skin, subcutaneous tissue, platysma muscle, and other layers was made to expose the sternocleidomastoid muscle. The sternocleidomastoid muscle was opened and fixed with a dilator to expose the carotid triangle. The carotid sheath was opened, and the common carotid artery, internal carotid artery, external carotid artery, superior thyroid artery, and sublingual nerve were marked with colored bands and fixed. Under the microscope, the blood vessels were subsequently blocked. An incision was made along the midline of the internal carotid artery before and after the bifurcation of the carotid artery. Plaques and inner intima were separated along with the interval between inner and middle intima. The inner intima of the carotid artery was severed at the interface of plaque segmentation. The segment of the internal carotid artery against the heart was segregated up and dragged out as much as possible by intima stripers. Moreover, heparin saline wash was repeatedly used to clean the floats and tiny plaque from the blood vessels after closing the 6-0 vascular suture.

**Figure 2:**
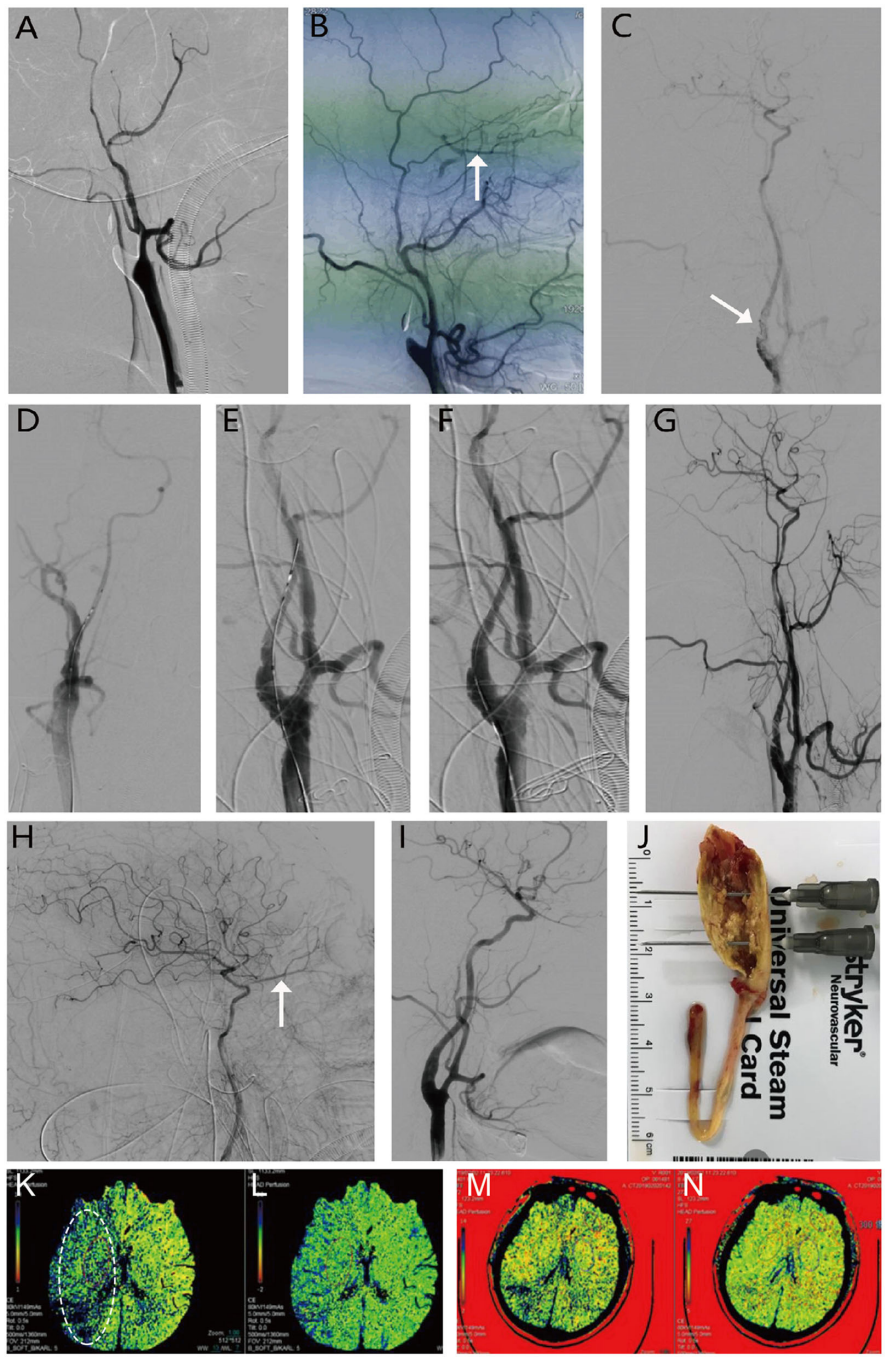
Procedure of open carotid endarterectomy (CEA) + internal carotid artery stent implantation (CAS) for right internal carotid artery occlusion. **(A, B)** Show occlusion of the right internal carotid artery, with (white arrow) indicating the backward opening of the ophthalmic artery, and the external carotid artery supplying blood to the internal carotid artery. **(C)** Shows that the internal carotid artery was open after CEA, but proximal stenosis is present (dashed white arrow) with poor intracranial perfusion. **(D, E)** Illustrate the procedure for CAS. **(F–H)** Show that after stent implantation, local stenosis is relieved, intracranial blood perfusion is significantly improved, and positive blood flow (white arrow) is restored to the ophthalmic artery. **(I)** Shows results of digital subtraction angiography at 6 months postoperatively. Further recovery of the vascular structure is observed without stenosis. **(J)** Shows intraoperative CEA with the removal of the internal carotid artery plaque and polarized thrombus, measuring ~10 cm. **(K–N)** Preoperative and postoperative perfusion CT. Compared with MTT in (**K**, white circle) and delayed TTP in **(L)** preoperatively, postoperative perfusion CT showed improvements in the **(M)** mean transit time (MTT) and **(N)** time to peak (TTP) after recanalization.

From the perspective of the guiding catheter, an appropriate thread was chosen, and a tube was inserted through the distal internal carotid artery occlusion. Angiography was performed again to ensure that the vascular cavity was reached. The internal carotid artery stent was properly expanded to open the distal occlusion of the blood vessels. The final imaging study was used to determine the patency of the occluded segment and intracranial blood perfusion. Blood pressure was reduced moderately after opening the occlusion to avoid hyperperfusion. The blood vessels and muscles of the neck were sutured successively, and the skin was sutured subcutaneously without placing a drainage tube (Figure 2 for a typical case).

### Postoperative management

Blood pressure was strictly controlled for at least 1 week after vascular opening to be 10–20% lower than the baseline blood pressure (i.e., the mean blood pressure at 3 days preoperatively). Patients' cognitive function, pupils, physical activity, and mental state were observed, and moderate fluid replacement was provided. Brain MRI combined with diffusion-weighted imaging (DWI), a neck vascular US, a TCD study, and indicators of myocardial infarction were reviewed within 1 week postoperatively.

### Follow-up management

At 1 month, 3 months, 6 months, 1 year, 1.5 years, and 2 years postoperatively, we reviewed patients' general blood test results, biochemistry findings, blood coagulation, platelet aggregation rate, US examination of the neck vessels, TCD results, and other examinations; determined the KPS scores; and recorded the time of recurrent stroke. Total cerebral angiography and brain MRI + DWI were re-examined at 6 months, 1 year, and 2 years postoperatively.

### Statistical analysis

All normally distributed continuous variables were reported as mean ± standard deviation (SD), and categorical data were reported as percentages or frequencies for descriptive characteristics. We assessed the differences between the two groups using the *t*-tests or Mann–Whitney *U*-test for continuous variables and the χ^2^ or Fisher's exact tests for continuous and categorical variables. The statistical significance level was set at a *p*-value of ≤ 0.05, and all data were analyzed using IBM SPSS Statistics for Windows, version 22.0 (IBM Corp., Armonk, New York, USA) for statistical analysis.

## Results

### Patient demographics and outcomes

A total of 22 patients (15 men and 7 women) were finally included in the study, ranging in age from 42 to 77 years (average age, 62.91 ± 8.34 years). Patients were categorized into two groups (success group and failure group) based on recanalization or not. Of all 22 patients, 19 were successfully recanalized with a success rate of 86.36% and 3 were not (Table 1). Three cases of hybrid surgery recanalization failure were transferred for EC-IC bypass and had good neurological recovery. Age, sex, smoking, diabetes, hypertension, heart disease, and duration of occlusion did not differ significantly between the success and failure groups (*P* > 0.05; Table 1). All the patients had an atherosclerotic plaque in the imaging analysis, which was confirmed by preoperative US and HRMRI. Preoperative and intraoperative DSA shows that occlusion length was less significant in the failure than in the success group (*P* > 0.999). Significantly different classification of lesions was observed between groups.

**Table 1:** Preoperative and postoperative factors related to the recanalization rate.

**Factors**	**Success group (*n* = 19)**	**Failure group (*n* = 3)**	***P*-value**
Age	62.5 ± 8.9	65.3 ± 3.2	0.6
**Sex**			>0.999
Male	13 (68.4%)	2 (66.7%)	
Female	6 (31.6%)	1 (33.3%)	
Smoking	12 (63.2%)	2 (66.7%)	>0.999
**Past medical history**			
Diabetes	11 (57.9%)	1 (33.3%)	0.571
Hypertension	10 (45.5%)	2 (66.7%)	>0.999
Heart disease	5 (26.3%)	1 (33.3%)	>0.999
**Duration of occlusion**			>0.999
<6 months	7 (36.8%)	1 (33.3%)	
≥6 months	12 (63.2%)	2 (66.7%)	
**Length of occlusion**			>0.999
<5 cm	8 (42.1%)	1 (33.3%)	
≥5 cm	11 (57.9%)	2 (66.7%)	
**Reverse ophthalmic artery blood flow in ICA**			0.038^*^
Yes	18 (94.7%)	1 (33.3%)	
No	1 (5.3%)	2 (66.7%)	
**Postoperative complications**			
Ischemic events	1 (5.3%)	0	
Hyperperfusion	1 (5.3%)	0	
Carotid-cavernous fistula	2 (10.5%)	1 (33.3%)	
Death	0	0	

^*^p ≤ 05, which is statistically different.

Interestingly, reverse ocular artery blood flow in ICA was found in 19 patients (86.4%), with 18 successful recanalizations and 1 failure. The success rates of CICAO with or without reverse ophthalmic artery blood flow in ICA were 94.7 and 33.3% in the success group and the failure group, respectively (*P* = 0.038). Notably, three patients had occlusions without reverse ophthalmic artery blood flow in ICA that exceeded the distal part of the ophthalmic artery and one underwent successful recanalization. The recanalization rate of this sub-group was 33.33%.

**Figure 3:**
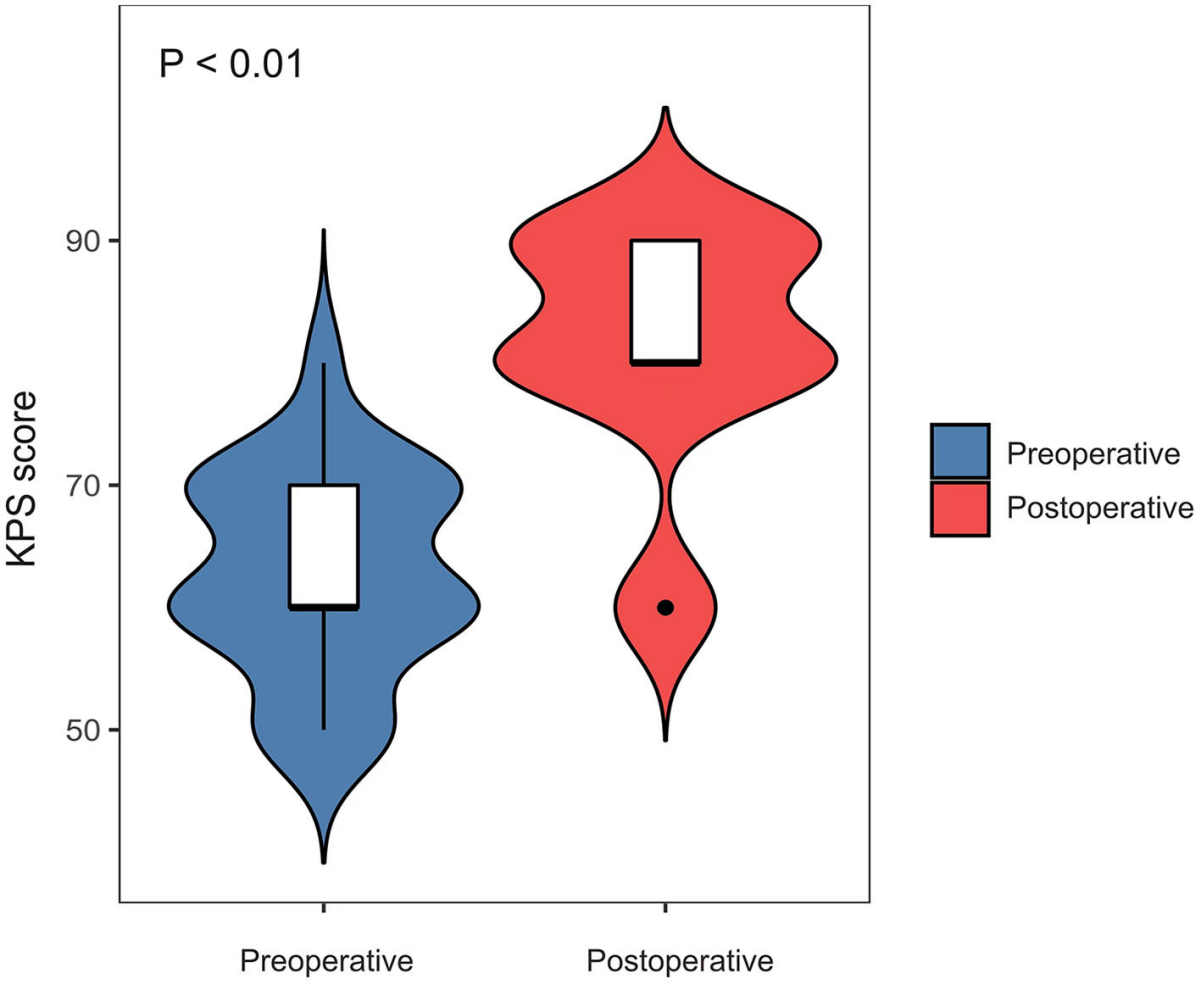
Comparison of preoperative vs. postoperative changes for a KPS score in the success group.

The postoperative complications and outcomes are shown in Table 1 and Figure 2. After successful recanalization, ischemic events occurred in one patient. One patient developed hyperperfusion syndrome. Three patients developed a carotid-cavernous fistula after the CEA procedure. After the proper treatment, both patients recovered completely. The average KPS scores of the 19 patients were 62.63 ± 8.72 preoperatively and 84.74 ± 5.67 postoperatively (*P* < 0.01; Figure 3).

## Discussion

Previous studies have shown that there are many factors that could influence the recanalization rate of CICAO. The morphology of the lesion also needs to be considered; according to the Hasan et al. classification, types A/B, C, and D occlusions have 95–100%, 45–50%, and 25–30% of recanalization success rates, respectively (8). Types A and B have higher recanalization rates and lower complication rates compared to type C and type D. Visualization of the initial segment of the ICA at the common carotid artery bifurcation, combined with the presence of supraclinoid filling, makes CAS suitable for occlusion types A and B (13). To seek better treatment, Pinter et al. (10), Shih et al. (14), and Zhang et al. (15) attempted to apply hybrid surgery to the treatment of CICAO and achieved success. In 2013, Shih et al. addressed that the occluded internal carotid artery was successfully opened for three patients through hybrid surgery with CEA + CAS (14). Subsequently, some centers have performed relevant clinical research and achieved particular success (6, 11, 16). Hybrid surgery was proven to be a feasible and efficient treatment and increased the success rate of recanalization in patients with CICAO (12). In the present study, we found that hybrid surgery has apparent advantages in recanalizing chronic internal carotid artery occlusion with a high recanalization rate, and reverse ophthalmic artery blood flow in ICA preoperatively is a predictor of successful recanalization.

Our study shows that hybrid surgery has apparent advantages in treating CICAO with ineffective endovascular treatment. Among the 22 patients in this study, 19 had a successful opening of the internal carotid artery occlusion, and the recurrence rate of occlusion was 86.4%, which was higher than in previous studies (15, 16). After surgery, one patient developed a small frontal infarction; infarction incidence was only 5.3% with no death. We suggest that implementing CEA first in the recanalization of the internal carotid artery occlusion and excision of the plaque and polarized thrombus of the internal carotid artery occlusion as much as possible are necessary to provide a proper operative channel for performing CAS by shortening the distance between the distal step in the opening, and surgeons are more likely to find the actual cavity and the success rate of opening can be improved, especially for type C internal carotid artery occlusion in Hasan classification. Hybrid surgery can also effectively remove the plaque and its components in the neck, reduce the incidence of intra- and postoperative infarctions, and reduce the restenosis rate.

In this case group, the average KPS score of 19 patients who underwent opening of the occlusion before surgery is 62.63 ± 8.72, and the average KPS score 6 months after surgery is 84.74 ± 5.67, with a bilateral comparison *P*-value of <0.001. There are statistical differences in the preoperative and postoperative KPS scores, suggesting that recanalization of the internal carotid artery occlusion could improve patients' living ability and cognitive function. Most scholars suggest that internal carotid artery recanalization, as a secondary prevention surgery, can reduce the recurrence rate of stroke, reducing the death and disability rate. However, it is unclear whether recanalization can improve a patient's quality of life. This study compares and analyzes the preoperative and postoperative KPS scores and clarifies the role of recanalization of internal carotid artery occlusion in improving patients' living ability and cognitive function.

Previous studies found that prior neurological events, stump morphology, distal ICA reconstitution via contralateral injection, and distal ICA reconstitution at communicating or ophthalmic segments influence successful recanalization. Our results indicate that the reverse ocular artery blood flow in ICA preoperatively is a predictor of successful recanalization. If the occlusion does not exceed the distal part of the ophthalmic artery, the reverse ocular artery blood flow in ICA can be observed in preoperative or intraoperative DSA. In this event, the possibility of successful recanalization is higher than that exceeded the distal part of the ophthalmic artery. Among the 22 patients, 3 had occlusions that exceeded the distal part of the ophthalmic artery, and 1 underwent recanalization; thus, the recanalization rate of this group was 33.33%. Among the 22 patients, 19 had occlusions that did not exceed the distal part of the ophthalmic artery, and one did not undergo open CEA; thus, the recanalization rate of occlusion for this group was 94.74%. The recanalization rate was statistically significantly different between the groups of the abovementioned patients (*P* = 0.038). Other studies have shown that the occlusion length, involved segment, occlusion time, patient age, etc. are related to the recanalization rate (9, 14). However, no statistical difference in the recanalization rate was found on gender, age, occlusion period, and length in our study.

## Limitations

Admittedly, our research also has some defects. First, this study is a retrospective study and not a randomized controlled study, which may lead to bias in patient selection and registration. Second, the number of cases in our study is smaller, which lacks comparative studies and short follow-up duration. Therefore, a larger sample size would have been needed to mitigate these effects. Finally, there may be some bias due to the different treatment timing of the symptomatic chronic occlusion of the carotid artery.

## Conclusion

Hybrid surgery for complex CICAO is safe and effective with a high recanalization rate. Recanalization of an internal carotid artery occlusion can improve patients' living ability and cognitive function. The recanalization rate of occlusions correlates with the occluded segment; thus, determining whether the occluded segment accumulates above the ophthalmic artery can be important in the preoperative evaluation of whether the artery can be opened.

## Data availability statement

The original contributions presented in the study are included in the article/supplementary material, further inquiries can be directed to the corresponding authors.

## Ethics statement

Ethical review and approval was not required for the study on human participants in accordance with the local legislation and institutional requirements. The patients/participants provided their written informed consent to participate in this study.

## Author contributions

WZ and JC were involved in the study design. TZ and YC were involved in writing the original draft and modifying the manuscript. LW and YM were involved in data curation. YF and GL were involved in the formal analysis. All authors have read and approved the final study protocol.
